# Officers of the American Medical Association

**Published:** 1878-08-20

**Authors:** 


### OFFICERS OF THE AMERICAN MEDICAL ASSOCIATION

APPOINTED AT ITS LATE MEETING:

President—Theophilus Marvin, MD, of Indiana.

Vice-Presidents—A J Fuller, MD, of Maine; W F Westmoreland, MD, of Georgia; John Morris, MD, of Maryland; John H Murphy, MD, of Minnesota.

Treasurer—Richard J Dunglison, MD, of Pennsylvania.

Librarian—Wm Lee, MD, of the District of Columbia.

Committee of Library—John Eliot, MD, of the District of Columbia.

Next place of meeting—Atlanta, Georgia.

Time of meeting—The first Tuesday in May, 1879.

Assistant Secretary—Scott Todd, MD, of Atlanta, Georgia.

Committee on Arrangements—Drs. J P Logan, chairman; H V M Miller, G G Crawford, H L Wilson, J F Alexander, J M Johnson, Charles Pinckney, V H Talliaferro, J T Johnson, of Atlanta, Georgia.

Committee on Prize Essays—Drs. Robert Battey, of Rome, Ga; J G Westmoreland, of Atlanta, Ga; Wm A Love, of Atlanta, Ga; Robert Ridley, of Atlanta, Ga; Henry F Campbell, of Augusta, Ga; J H Van Deman, of Chattanooga Tenn.

Committee on Publication—Drs. Wm B Atkinson, chairman; T M Drysdale, A Fricke, S D Gross, C Wister, R J Dunglison, of Penn; and Wm Lee, of the District of Columbia.

